# *Purple acid phosphatase 10c* encodes a major acid phosphatase that regulates plant growth under phosphate-deficient conditions in rice

**DOI:** 10.1093/jxb/eraa179

**Published:** 2020-04-09

**Authors:** Suren Deng, Linghong Lu, Jingyi Li, Zezhen Du, Tongtong Liu, Wenjing Li, Fangsen Xu, Lei Shi, Huixia Shou, Chuang Wang

**Affiliations:** 1 Microelement Research Center, College of Resources & Environment, Huazhong Agricultural University, Wuhan, P. R. China; 2 Key Laboratory of Arable Land Conservation (Middle and Lower Reaches of Yangtze River), MOA, Huazhong Agricultural University, Wuhan, P. R. China; 3 Institute of Horticulture, Zhejiang Academy of Agricultural Sciences, Hangzhou, P. R. China; 4 State Key Laboratory of Plant Physiology and Biochemistry, College of Life Sciences, Zhejiang University, Hangzhou, P. R. China; 5 University of Nottingham, UK

**Keywords:** Acid phosphatase, native promoter, organic phosphate, overexpression, Pi deficiency, rice, root

## Abstract

Whilst constitutive overexpression of particular acid phosphatases (APases) can increase utilization of extracellular organic phosphate, negative effects are frequently observed in these transgenic plants under conditions of inorganic phosphate (Pi) sufficiency. In this study, we identified rice purple acid phosphatase 10c (OsPAP10c) as being a novel and major APase that exhibits activities associated both with the root surface and with secretion. Two constructs were used to generate the *OsPAP10c*-overexpression plants by driving its coding sequence with either a ubiquitin promoter (UP) or the *OsPAP10c*-native promoter (NP). Compared with the UP transgenic plants, lower expression levels and APase activities were observed in the NP plants. However, the UP and NP plants both showed a similar ability to degrade extracellular ATP and both promoted root growth. The growth performance and yield of the NP transgenic plants were better than the wild-type and UP plants in both hydroponic and field experiments irrespective of the level of Pi supply. Overexpression of APase by its native promoter therefore provides a potential way to improve crop production that might avoid increased APase activity in untargeted tissues and its inhibition of the growth of transgenic plants.

## Teaser

Overexpression of *OsPAP10c* driven by its native promoter enhances the growth and yield of rice in comparison both with the wild-type and with plants where overexpression is driven by a ubiquitin promoter.

## Introduction

With the increasing demands on agricultural production worldwide, phosphorus (P) is receiving more and more attention as a non-renewable resource (Gilbert, 2009). Although P is abundant in the Earth’s crust, plants can only take up the water-soluble inorganic phosphate (Pi) in the soil. As P diffuses slowly and is highly fixed in the soil, the concentration of Pi is usually very low, and not enough to support the farming systems in many countries. Consequently, to obtain high crop yields, chemical P fertilizer is widely used on agricultural land and has increased costs and caused environmental damage (Shen *et al.*, 2011). Developing crop varieties that are adapted to conditions of low-Pi stress therefore has an important role to play in developing sustainable agriculture and maintaining the global food supply.

Pi starvation has been reported to induce the synthesis of intra- and extracellular acid phosphatase (APase) isozymes in plants (Bozzo *et al.*, 2006). More specifically, intracellular acid phosphatases have been shown to be up-regulated after 2 d of Pi starvation, which coincides with a reduction in the levels of intracellular free Pi, and they are believed to be involved in recycling Pi from intracellular P metabolites (Bozzo *et al.*, 2006; Pratt *et al.*, 2009). In contrast, under prolonged conditions of Pi starvation, secreted acid phosphatases accumulate in the growing medium in order to degrade extracellular organic P (Po) compounds (Bozzo *et al.*, 2006). The major intracellular and secreted acid phosphatases have been determined in several plant species, and the majority of them are encoded by purple acid phosphatase genes (Miller *et al.*, 2001; Bozzo *et al.*, 2004; Veljanovski *et al.*, 2006; Liang *et al.*, 2010; Tran *et al.*, 2010b).

Purple acid phosphatases (PAPs; EC 3.1.3.2) are a family of binuclear metalloenzymes that show a purple or pink colour in solution (Olczak *et al.*, 2003). A wide range of phosphate esters and anhydrides can be hydrolysed by different PAPs (Tran *et al.*, 2010a). PAPs were first isolated from mammalian species and play a role in bone metabolism (Feder *et al.*, 2019a). Serum concentrations of PAPs are significantly increased in patients suffering from osteoporosis, and hence several inhibitors have been developed as therapeutic agents to treat this condition (Hussein *et al.*, 2018; Feder *et al.*, 2019b). Since their discovery in mammals, many PAP isoforms have also been purified from plant tissues (del Pozo *et al.*, 1999; Miller *et al.*, 2001; Bozzo *et al.*, 2004), and the physiological functions of a number of them have been characterized, most of which are related to P nutrition (Tran *et al.*, 2010b; Wang *et al.*, 2011; Robinson *et al.*, 2012; Lu *et al.*, 2016; Li *et al.*, 2017; Mehra *et al.*, 2017; Wu *et al.*, 2018). In Arabidopsis, AtPAP12 and AtPAP26 have been shown to be the predominant PAP isozymes that are secreted into the medium under Pi starvation (Tran *et al.*, 2010b; Ghahremani *et al.*, 2019). Mutations of *AtPAP12* and *AtPAP26* result in a significant reduction in the activity of secreted APases and hence in the ability to utilize extracellular Po (Robinson *et al.*, 2012). AtPAP12 and AtPAP26 can also be secreted into the cell wall and account for a substantial proportion of the APase activity localized to the root surface (Robinson *et al.*, 2012). However, mutation of *AtPAP10* results in a ‘white root’ phenotype and impaired growth, indicating that AtPAP10 is the major APase isoform associated with the roots in Arabidopsis (Wang *et al.*, 2011; Zhang *et al.*, 2014). Recently, two root-associated APase isozymes induced by Pi starvation in soybean, GmPAP1-like and GmPAP21, have been identified using a proteomic analysis of cell wall proteins (Wu *et al.*, 2018), and they have been shown to be involved in the adaptation of soybean roots to Pi starvation (Li *et al.*, 2017; Wu *et al.*, 2018). In addition to utilization of Po, NtPAP12 and OsPAP21b have been reported to regulate cell wall biosynthesis and root development (Kaida *et al.*, 2009; Mehra *et al.*, 2017).

As Po can account for 30–65% of the total P in soils, a number of acid phosphatases have been used to generate transgenic crops that have an increased utilization ability in order to improve growth performance under low-Pi conditions. In rice, overexpression of *OsPAP10a*, *OsPAP10c*, and *OsPAP21b* significantly increase APase activity and Po degradation (Tian *et al.*, 2012; Lu *et al.*, 2016; Mehra *et al.*, 2017). The extracellular utilization of ATP is significantly enhanced in hairy roots of transgenic beans with overexpression of *PvPAP3* or *GmPAP1-like* (Liang *et al.*, 2010; Wu *et al.*, 2018). In addition, in transgenic Arabidopsis with overexpression of *AtPAP10*, *AtPAP12*, and *AtPAP26*, growth is improved when plants are supplied with ADP and Fru-6-P (Wang et al., 2011, 2014). Overexpression of *AtPAP15* and *SgPAP23* (*Stylosanthes guianensis*), which show high phytase activities, facilitates the utilization of phytate-P and significantly improves the growth of transgenic plants (Wang *et al.*, 2009; Liu *et al.*, 2018). Transgenic soybean lines with overexpression of *AtPAP15* also exhibit improved yields when grown on acid soils (Wang *et al.*, 2009).

Although the overexpression of these *PAP* genes successfully increases the ability to degrade Po and improves plant growth under Pi-deficient conditions, the transgenic plants usually show either no response or inhibited growth performance under Pi-sufficient conditions (Wang *et al.*, 2014; Lu *et al.*, 2016; Liu *et al.*, 2018; Wu *et al.*, 2018). To avoid the negative effects of constitutive overexpression of *PAP* genes, in this study we increased the expression of *OsPAP10c* by means of its own promoter. It has previously been reported that *OsPAP10c* is a novel monocotyledon *PAP* gene that is specifically induced by Pi starvation in roots (Lu *et al.*, 2016). We found that mutants of *OsPAP10c* had significantly decreased activities of both root surface-associated and secreted APase, indicating that OsPAP10c is one of the major APase isoforms in rice. Compared with plants with constitutive overexpression of *OsPAP10c*, transgenic plants with *OsPAP10c* driven by the native promoter showed lower transcripts levels and APase activity in untargeted tissues. The growth performance and yield of the transgenic plants driven by the native promoter were better than those of the wild-type and of plants with constitutive overexpression in both hydroponic and field experiments irrespective of the level of Pi supply.

## Materials and methods

### Plant materials and growth conditions

Seeds of rice (*Oryza sativa*) cv Nipponbare were surface-sterilized with 1% nitric acid and germinated at 37 °C in the dark for 2 d. Uniform germinated seeds were cultured hydroponically using a solution containing 1.425 mM NH_4_NO_3_, 0.513 mM K_2_SO_4_, 0.998 mM CaCl_2_, 1.643 mM MgSO_4_, 0.25 mM NaSiO_3_, 0.009 mM MnCl_2_, 0.075 μM (NH_4_)_6_Mo_7_ O_24_, 0.019 μM H_3_BO_3_, 0.155 μM CuSO_4_, 0.152 μM ZnSO_4_, and 0.125 mM EDTA-Fe(II), pH 5.5. For the Pi-sufficient treatment (+Pi) the nutrient solution also contained 0.323mM NaH_2_PO_4_; the solution without NaH_2_PO_4_ represented the Pi-deficient treatment (–P).The seedlings were grown in a greenhouse under a 12/12-h photoperiod (200 µmol photons m^–2^ s^–1^) at 32/24 °C and 60% relative humidity.

For ATP degradation and utilization experiments, plants were grown in +P media for 15 d, after which half of the seedlings were treated with 0.1 mM ATP for a further 10 d.

For analysis of agronomic traits, plants were grown in an experimental field where they received either 80 kg ha^–1^ phosphate fertilizer (high-P treatment, HP) or no fertilizer (low-P treatment, LP). The properties of the soil were as follows: pH 7.01; organic matter, 9.03 g kg^–1^; available nitrogen, 76.38 mg kg^–1^; available potassium, 195.33 mg kg^–1^; and effective phosphorus, 6.11 mg kg^–1^. The following agronomic traits were determined at maturity: plant height, tiller number, seed-setting rate, 1000-grain weight, grain number per plant, and grain yield per plant. Seed-setting rate was calculated as the ratio of solid grains to total grains on the spikes.

### Vector construction and plant transformation

To create *OsPAP10c* CRISPR lines, a 20-bp sgRNA targeting the first exon of *OsPAP10c* was cloned into the CRISPR-Cas9 expression vector *pRGEB31* according to the method described by Xie *et al.* (2014). *OsPAP10c*-overexpression plants driven by a ubiquitin promoter (UP) were obtained as described by Lu *et al.* (2016). In addition, the full-length cDNA of *OsPAP10c* was cloned into the vector *pCAMIA1300* using the *OsPAP10c*-native promoter (NP) to drive its coding sequence (CDS). Transgenic plants were generated by *Agrobacterium*-mediated transformation as reported previously (Tian *et al.*, 2012).

To construct the *CaMV 35S::OsPAP10c* transformation vector, the CDS of *OsPAP10c* was amplified using a High-Fidelity PCR Kit (CWBIO) with the forward primer 5´-CGGAGCTCGGTACCCATGGGGATGCTGCGGTGG-3´ and reverse primer 5´-CTCTAGAGGATCCCCCTACATCGTCGTTGGTGGGC-3´. The amplified CDS was subsequently cloned into the pCAM1300-mod vector using a GBclonart Seamless Cloning Kit (GBI, Suzhou, China) according to the manufacturer’s instructions. The construct was transferred into *Agrobacterium tumefaciens* strain GV3101 and transformed into the Arabidopsis *pap10* mutant using the floral dip method (Clough and Bent, 1998). The resultant transgenic plants were confirmed by their resistance to hygromycin and PCR analysis.

### Southern blot analysis of transgenic plants

For Southern blot hybridization analysis, 10 μg of genomic DNA was digested with the *EcoR* I restriction enzyme, separated on a 0.8% agarose/TAE gel, and capillary-blotted onto a nylon membrane. The *hygromycin phosphotransferase* (*hpt*) probe and *OsPAP10c* probe were labeled with digoxigenin and the membranes were hybridized with each probe separately. The membranes were visualized using a multifunctional image analyser (FLA-1500, FUJIFILM).

### Quantitative real-time PCR

Total RNA was extracted from the tissues of rice plants using TRIzol reagent (ThermoFisher Scientific). Samples of 2 μg of total RNA were used to synthesize cDNA using a High-Capacity cDNA reverse-transcription kit (ThermoFisher Scientific). qRT-PCR assays were performed with SYBR Green Real-Time PCR Master Mix reagent (Takara) on an Applied Biosystems Quantstudio^TM^ Real-Time PCR System. The rice actin gene (*OsACTIN*) was used as the internal reference and relative expression levels were calculated using the 2^–ΔΔ*C*T^ method (Livak and Schmittgen, 2001). The sequences of the *OsACTIN* primers were as follows: 5´-GACGGACGCACCCTGGCTGA-3´ and 5´-TGCTGCCAATTACCATATACC-3´. The sequences of the *OsPAP10c* primers were as follows: 5´-AGCCGTTCTGGTACTCCGTCAAG-3´ and 5´-CGCGCATCGTCTCCCCCTCCAT-3´. The sequences of the *OsPAP10a* primers were as follows: 5´-GAGATAGATTTTGCCCCAGAACT-3´ and 5´-AGCTTCAAGCCACTTGTACTGAG-3´.

### Protein extraction and quantification of APase activity in plant tissues

Total protein was extracted from root and leaf tissues in frozen extraction buffer as described by Lu *et al.* (2016). The protein content was quantified using Coomassie Blue in assay reagent. For quantification of tissue APase activity, 1 µg (roots) or 10 µg (leaves) of total protein were incubated with 600 µl of 10 mM p-nitrophenyl phosphate (pNPP) in a reaction mixture containing 50 mM sodium acetate, pH 5.5. Reactions were incubated at 25 ºC for 30 min and were then stopped by the addition of 1.2 ml of 1 M NaOH. The absorbance was determined at 410 nm using a microplate assay (Tecan). The APase activity was expressed as μmol pNP min^–1^ mg^–1^ protein.

### Protein extraction from culture media and in-gel APase profiling

Rice calli were liquid-cultured according to the method described in detail by Lu *et al.* (2016). In brief, the calli were cultured with Pi-sufficient and -deficient R2S solutions for 7 d, and the liquid culture media were then collected. Proteins were concentrated and isolated using ultrafiltration centrifuge tubes (Millipore) with a 10-kDa cut-off at 4 °C according to the manufacturer’s instructions. The protein contents were quantified using Coomassie Plus (Bradford) Assay Kit (ThermoFisher Scientific). In-gel APase profiling was carried out as described by Wang *et al.* (2011). The proteins were separated on a 10% non-reducing PAGE gel at 4 °C. The gels were gently washed in cold distilled water to remove salt (10 min per wash and a total of six washes). The gels were then washed twice (15 min each) in a buffer containing 50 mM sodium acetate (pH 4.9) and 10 mM MgCl_2_. After equilibrium with the buffer, the gels were stained for APase activity in buffer containing 0.5 mg l^–1^ Fast Black potassium salt and 0.5 mg l^–1^ ß-naphthyl acid phosphate at 37 °C. The gels were then washed in distilled water and imaged.

### Activity assays for root-associated APase

*In vivo* root activity staining of acid phosphatase was performed as previously described (Tian *et al.*, 2012). Briefly, the roots of seedlings were excised and incubated with a 5-bromo-4-chloro-3-indolyl-phosphate (BCIP)-agar overlay solution containing 50 mM Na-acetate, 10 mM MgCl_2_, 0.5% agar, 0.05 mg ml^−1^ BCIP, pH 5.3, for 2 h at 25 °C for the visualization of the activity of root secreted acid phosphatases. The BCIP solution is a colorless acid phosphatase substrate and the activity is visualized as the blue color that results from its hydrolysis.

For quantification of root-associated APase activities, individual seedling was rinsed in distilled water and transferred to centrifuge tubes containing culture medium with 10 mM pNPP (pH 5.5). The seedlings were then placed in an incubator at 37 °C for 30 min. The reaction was stopped with 1 M NaOH and the absorbance was determined at 410 nm using a microplate assay (Tecan). APase activity was normalized to the fresh weigh of the roots (units g^–1^ FW) or expressed per seedling of Arabidopsis (nmol pNP min^–1^ per seedling).

### Measurements of tissue Pi concentration and total P content

Pi concentrations were measured using the methods described previously by Lu *et al.* (2016). Briefly, 25 mg of fresh tissue samples were homogenized with 25 µl 5 M H_2_SO_4_ and 1.5 ml distilled water. After centrifugation at 11 180 *g* at 4 °C the supernatant was collected. The supernatant was diluted and mixed with malachite green reagent (19.4 mM H_3_BO_3_, 27.64 mM (NH_4_)_6_MO_7_O_24_.4H_2_O, 2.38 M H_2_SO_4_, 627.5 μM malachite green, and 0.1% polyvinyl alcohol) at a 3:1 ratio and absorbance at 650 nm was measured after 30 min using a microplate assay (Tecan). The Pi concentration was calculated based on a standard curve generated using varying concentrations of KH_2_PO_4_. Free Pi released from ATP in the nutrient solutions was measured directly using the malachite green reagent described above.

For measurement of total P content, 150 mg of dried plant tissue was pre-digested in glass tubes with concentrated sulfuric acid overnight. The tubes were then heated to 120 °C for 1 h and 4–5 drops of 30% H_2_O_2_ were added every 30 min until the solution turned colourless. The digestion was continued for an additional 30 min, and then the total P concentration was determined by molybdenum blue colorimetry. In this study, we define the P-use efficiency as the amount of biomass produced by a given amount of P content in the tissue, according to Liao and Yan (1999)

### Statistical analysis

SPSS Statistics Base software (version 22) was used for statistical analysis. Significant differences were evaluated using one-way ANOVA and Turkey’s test.

## Results

### Expression of *OsPAP10c* is specifically induced by Pi starvation in roots

**Fig. 1.**
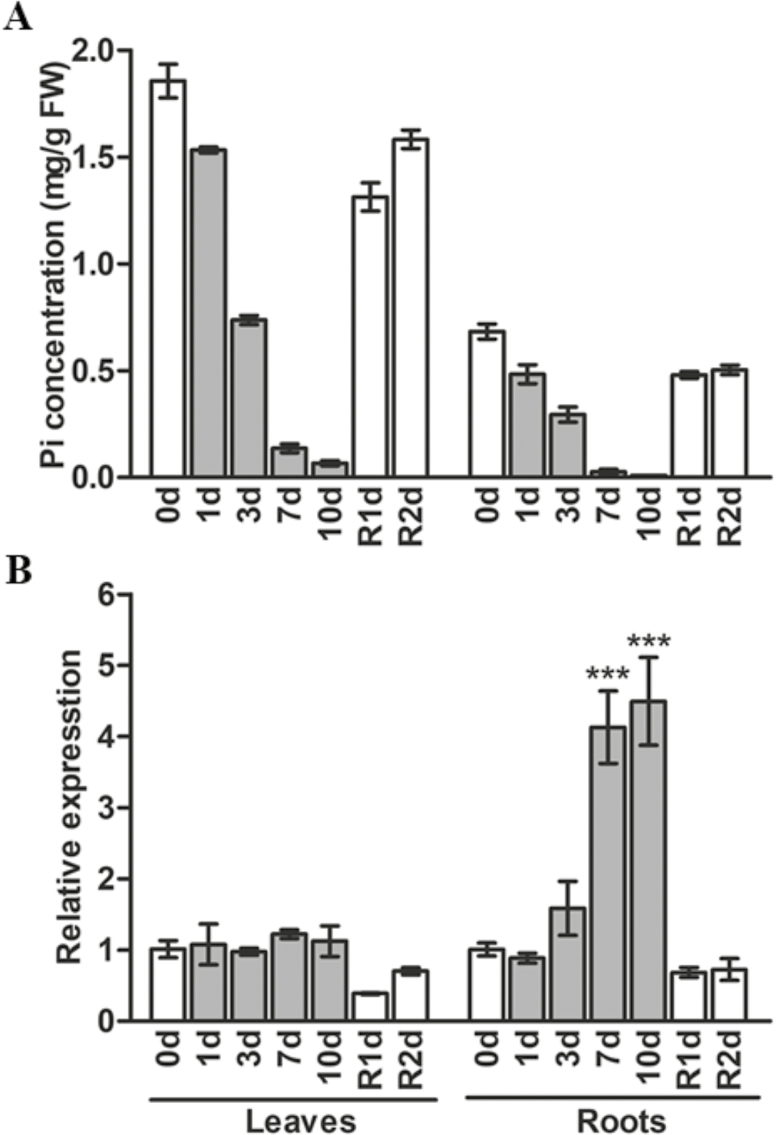
(A) Pi concentration and (B) expression of *OsPAP10c* in leaves and roots of rice during a period of P starvation followed by resupply. Germinated seeds were grown in normal nutrient solution for 10 d and then transferred to solution without Pi for 10 d, followed by 2 d recovery (R) in normal solution. RNA was extracted for quantitative RT-PCR and *OsPAP10c* expression was normalized to that of *OsACTIN*. Data are means (±SEM) of three replicates. Significant differences compared with the control (0 d) were determined using Turkey’s test (***P<0.001).

To determine the expression of *OsPAP10c* in rice under Pi-starvation conditions, a time-course of transcript levels was extracted from published RNA-seq data (Secco *et al.*, 2013), which showed that transcripts increased in roots within 3 d of a Pi-deficient treatment (Supplementary Fig. S1 at *JXB* online). By 7 d of treatment the transcript level had increased by more than 10-fold, and this was maintained at 21 d. However, after a recovery period of resupply of Pi for only 1 d the expression of *OsPAP10c* had decreased back to the same level as the control roots. In contrast to the roots, there was no effect on transcript levels in response to Pi starvation even after 21 d in the shoots. To confirm these RNA-seq results, we used qRT-PCR to determine the expression of *OsPAP10c* at different time-points during Pi starvation. The concentrations of Pi in both the roots and leaves steadily decreased with time in response to the lack of supply (Fig. 1A). Resupply of Pi in the nutrient solution significantly increased the concentrations in both the roots and shoots. The decrease in Pi concentration in the roots was accompanied by a significant increase in the expression of *OsPAP10c* after 7 d and 10 d of the starvation treatment, but expression returned to the control level after 1 d of resupply (Fig. 1B). In contrast, the expression of *OsPAP10c* was maintained at a low level in the shoots irrespective of the Pi supply and the changes in internal concentration. These results therefore showed that the expression of *OsPAP10c* was specifically induced by Pi starvation only in the roots.

### Mutations of *OsPAP10c* decrease the activities of root-associated and secreted acid phosphatase

**Fig. 2.**
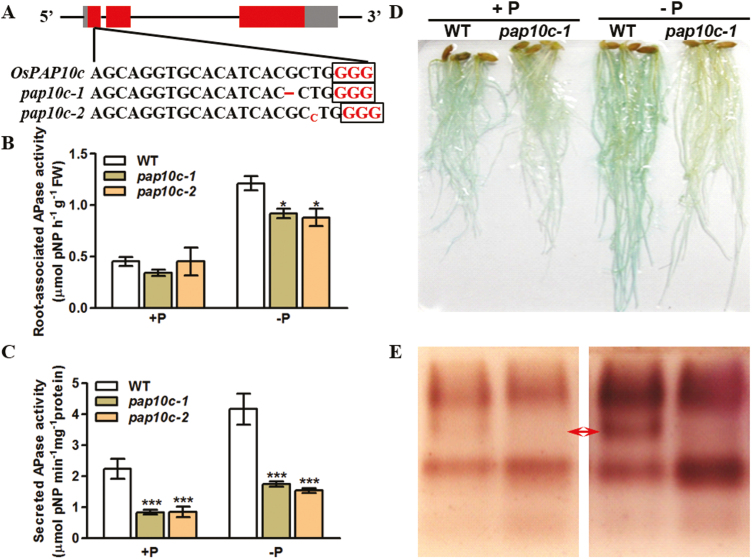
APase activity in rice *Ospap10c* mutants. (A) Schematic diagram of deletion mutations at the target sites in two representative knock-out lines generated by CRISPR/Cas9 technology. The sgRNA target sequence is shown and the protospacer adjacent motifs are indicated by the boxes. (B) Root surface-associated APase activity of the wild-type (WT) and *pap10c* mutants under +P and –P conditions. (C) Secreted APase activity of the WT and *pap10c* mutants under +P and –P conditions. (D) BCIP staining of the WT and the *pap10c-1* mutant under +P and –P conditions. (E) In-gel APase activity assay of secreted proteins extracted from suspension cells of the WT and *pap10c-1* mutant. The arrows indicate that the absence of a specific APase isoform in the mutant under Pi-deficient conditions.

**Fig. 3.**
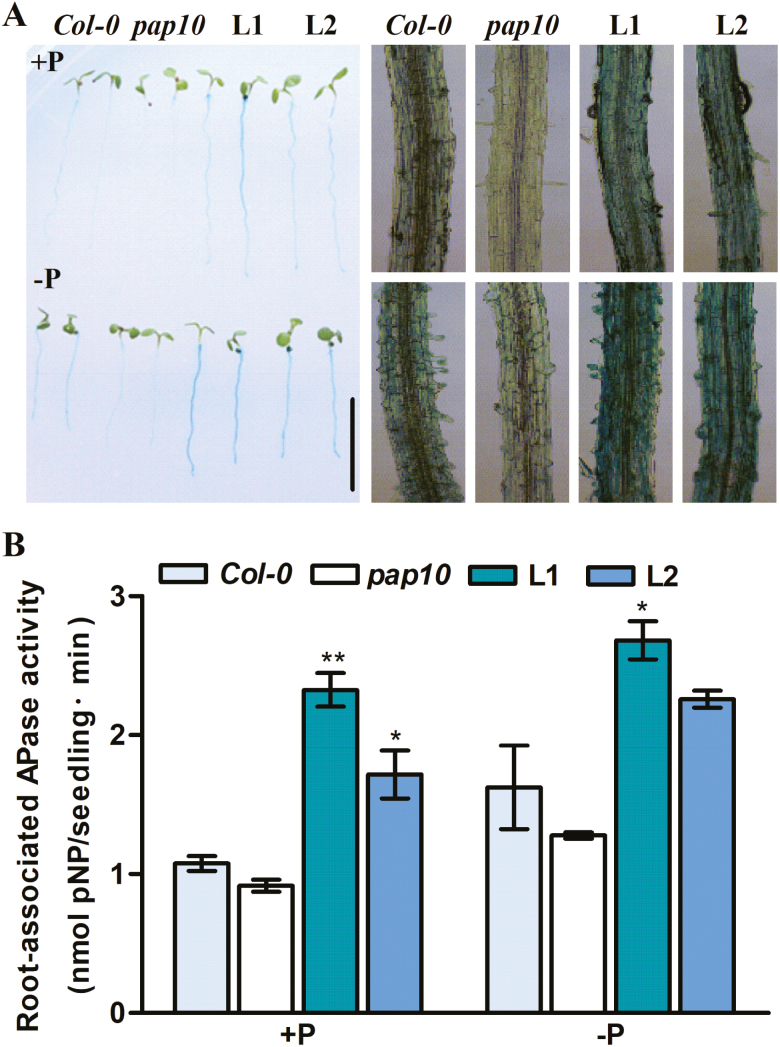
Root-associated APase activity in Arabidopsis Col-0, the *Atpap10* mutant, and *OsPAP10c* complementary lines in the *Atpap10* background (L1, L2) under +P and –P conditions. (A) BCIP staining of roots of 7-d-old plants. The scale bar is 1 cm. (B) Quantification of the APase activity. Data are means (±SEM) of three replicates. Significant differences compared with Col-0 were determined using Turkey’s test (*P<0.05; **P<0.01).

To examine whether OsPAP10c is a major secreted acid phosphatase in rice, we transformed a CRISPR-Cas9 construct that targeted the first exon of *OsPAP10c* into the Nipponbare wild-type (WT) and identified two knock-out plants with frame shifts (*pap10c-1* and *pap10c-2*, Fig. 2A). Different patterns of acid phosphatase (APase) activity were observed in the mutants compared with the WT. The APase activities in the leaves and roots were similar between the mutants and the WT plants under both Pi-sufficient and -deficient conditions (Supplementary Fig. S2); however, the activities of both the root surface-associated and secreted APase were significantly decreased in the mutants under deficient conditions (Fig. 2B, C). To further determine the surface-associated APase activity, roots of *pap10c-1* were stained with BCIP. Pi starvation induced less staining in the roots of the mutant than in those of the WT under both Pi-sufficient and -deficient conditions (Fig. 2D). The white roots of *Ospap10c-1* were reminiscent of the Arabidopsis *pap10* mutant, encoding a root-associated PAP. We therefore transformed *OsPAP10c* into the *Atpap10* mutant and obtained two *OsPAP10c*-overexpression lines in the mutant background. As previously reported, the *Atpap10* mutant showed white roots under BCIP staining and had decreased root-associated APase activity compared with the WT under both Pi-sufficient and -deficient conditions (Fig. 3A, B). Overexpression of *OsPAP10c* in *Atpap10* fully complemented the white root phenotype and significantly increased the root-associated APase activity. The secreted proteins from suspension cells were purified and analysed by in-gel APase activity assays in order to determine whether OsPAP10c was present as a major APase isoform. Pi starvation induced a specific APase isoform in the WT that was absent in the *Ospap10c* mutant (Fig. 2E). Taken together, the results indicated that OsPAP10c is not only a major root surface-associated APase in rice but also that it is a major secreted isoform.

### The native promoter increases expression of *OsPAP10c*

**Fig. 4.**
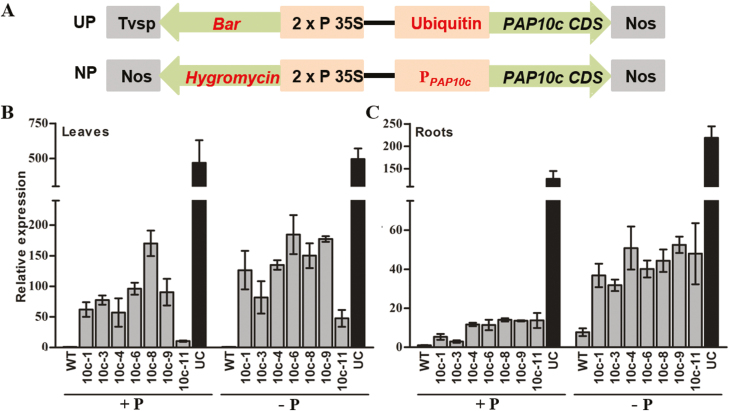
Relative expression levels of *OsPAP10c* in rice wild-type (WT) and transgenic plants. (A) Schematic diagram of the vectors with the constitutive ubiquitin promoter (UP) and the native promoter (NP). (B, C) Relative expression levels of *OsPAP10c* in the leaves and roots of the genotypes. The expression levels are relative to that of the wild-type in the +P treatment. Data are means (±SEM) of three replicates.

Although overexpression lines of *OsPAP10c* driven by a ubiquitin promoter (UP) have significantly enhanced APase activity in leaves and roots, the growth of the transgenic plants is not stimulated under both Pi-sufficient and -deficient conditions (Lu *et al.*, 2016). Because the expression of *OsPAP10c* is specifically induced by Pi starvation in roots, we made another construct using the *OsPAP10c* native promoter (NP) to drive its coding sequence and generated seven positive lines by *Agrobacterium*-mediated transformation (Fig. 4A). Southern blot analysis using the *hygromycin phosphotransferase* (*hpt*) probe detected one band in the lines NP-1, NP-6, and NP-9, and two or three bands in the other lines (Supplementary Fig. S3A). To further confirm the copy numbers in these transgenic lines, the *OsPAP10c* probe was used for Southern blotting. Interestingly, four bands were detected in the WT plants, indicating that there are several homologues of *OsPAP10c* in the rice genome (Supplementary Fig. S3B). Compared with the WT, an additional band was detected in the lines NP-1, NP-3, and NP-9, while the others contained more bands of *OsPAP10c*. Combining the information obtained from the two probes, it appeared that lines NP-1 and NP-9 were single-copy insertion events while the other lines were multiple-copy events.

The expression levels of *OsPAP10c* were measured in the leaves and roots of WT and the two types of transgenic plants under both Pi-sufficient and -deficient conditions. As expected, Pi starvation significantly induced the expression of *OsPAP10c* in the roots of all the genotypes, but the relative increase in the UP plants was less and not significant (Fig. 4C). Although expression was significantly increased in both the UP and NP plants compared with the WT, the level was much lower in the NP plants than in the UP plants in both the leaves and roots (Fig. 4B, C). The expression of *OsPAP10c* in the UP plants under both Pi-sufficient and -deficient conditions was nearly 500-fold and 127-fold that of the leaves and roots, respectively, of the WT plants. The mean expression level of *OsPAP10c* in the NP plants was only ~10% of that of the UP plants.

### Secreted APase activity and ATP degradation are increased in NP plants

To evaluate the effects of the increased copy number of *OsPAP10c* in rice, the transgenic plants were grown under Pi-sufficient and -deficient conditions. Compared with the WT, the growth of the UP plants was only enhanced under Pi-deficient conditions (Supplementary Fig. S4). In contrast, the growth of all the NP lines was better than the WT and the UP plants under both Pi-sufficient and -deficient conditions. Lines NP-1, NP-6, and NP-9 showed the highest FW and were selected for further analysis. APase activities were determined under different Pi supply conditions. APase activities were significantly increased in the leaves and roots in the UP plants under both Pi-sufficient and -deficient conditions (Supplementary Fig. S5). In contrast, under both conditions the APase activity of leaves was similar between the NP plants and the WT plants. Whilst the activity in the roots was higher in the NP plants than in the WT, it was lower than that of the UP plants, especially under Pi-sufficient conditions.

**Fig. 5.**
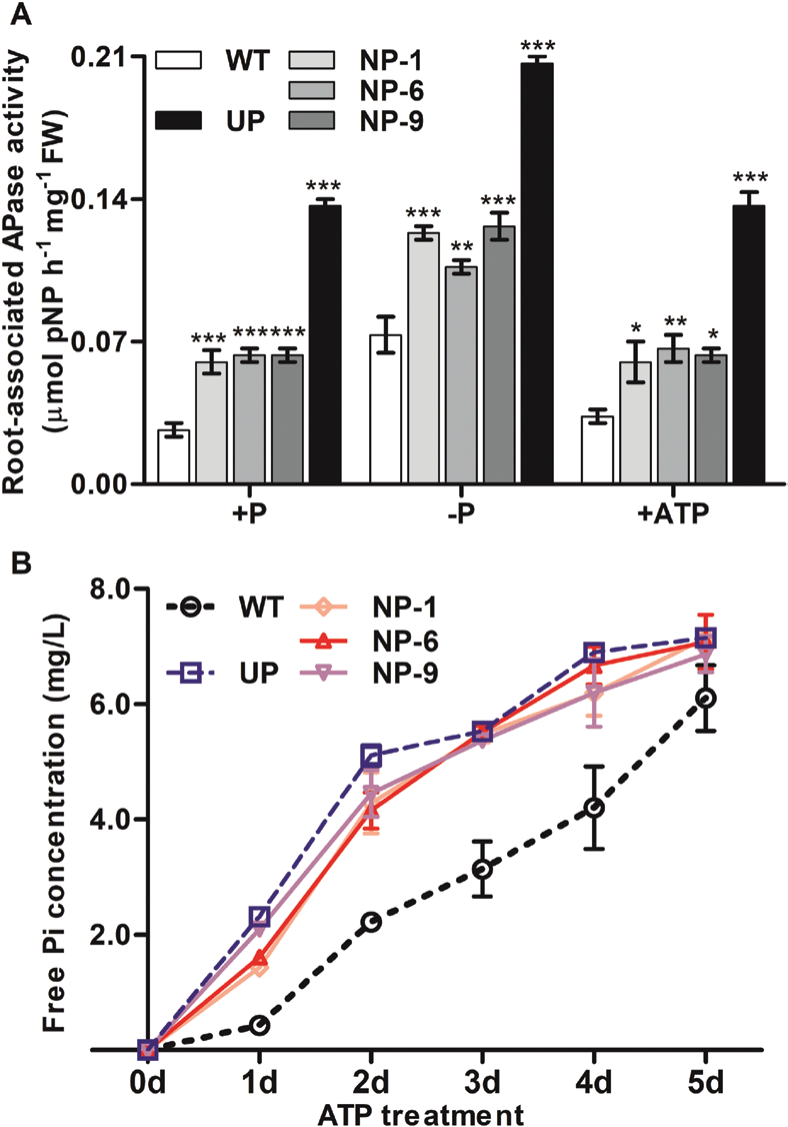
(A) Root-associated APase activity of the rice wild-type (WT) and of transgenic plants with *OsPAP10c* driven by the constitutive ubiquitin promoter (UP) or the native promoter (NP) grown either with or without Pi, or with ATP as the only source of P. (B) Concentration of free Pi in the growth solution for plants grown with ATP. Germinated seeds were grown in normal nutrient solution for 15 d and then transferred to solution either with or without Pi, or containing 0.1 mM ATP as the only source of P. Root-associated APase activities were measured after 10 d treatments. Data are means (±SEM) of three replicates. Significant differences compared with the WT were determined using Turkey’s test (*P<0.05; **P<0.01; ***P<0.001).

With regards to the activity of secreted APase, overexpression of *OsPAP10c* in the UP plants increased the activity by 7-fold in comparison to the WT under Pi sufficient-conditions (Fig. 5A), whilst there was only a 2-fold increase in the NP plants. Pi starvation significantly induced APase activity in all the genotypes, and levels were 3- and 1.5-fold greater in the UP and NP plants, respectively, compared with the WT. When ATP was used as the only P source, the activity of secreted APase was 2- and 5-fold greater in the UP and NP plants, respectively, compared with the WT. As previously reported, the UP plants released more Pi from ATP during the first 4 d of treatment compared with the WT (Fig. 5B; Lu *et al.*, 2016). Interestingly, a similar pattern for ATP degradation was observed between the NP and UP plants.

### Plant growth and accumulation of P are increased in NP plants

**Fig. 6.**
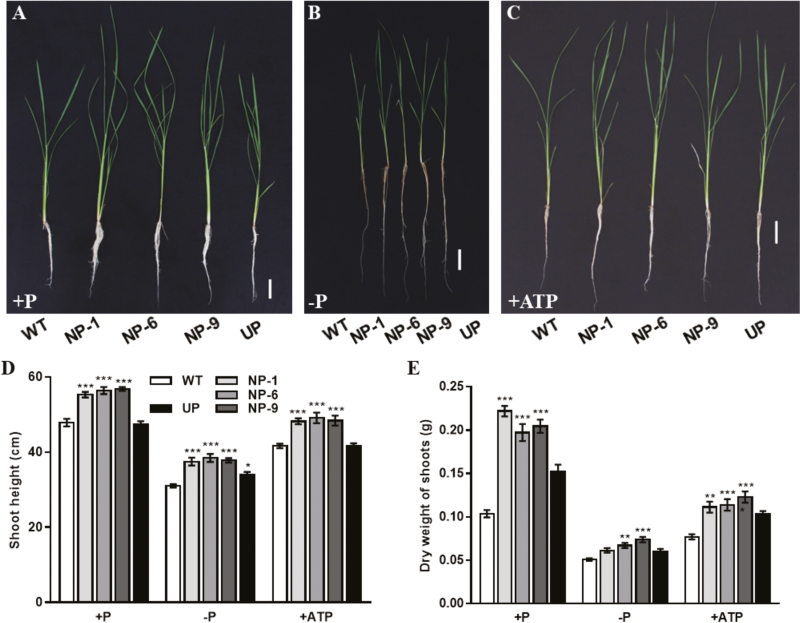
Phenotypes and growth of wild-type (WT) rice and of transgenic plants with *OsPAP10c* driven by the constitutive ubiquitin promoter (UP) or the native promoter (NP) grown either with or without Pi, or with ATP as the only source of P. Germinated seeds were grown in normal nutrient solution for 15 d and then transferred to different solutions for 10 d. (A–C) Phenotypes of seedlings under the different treatments. The scale bars are 5 cm. (D) Shoot height and (E) dry weight of the genotypes under the different treatments. Data are means (±SEM) of six replicates. Significant differences compared with the WT were determined using Turkey’s test (*P<0.05; **P<0.01; ***P<0.001).

**Fig. 7.**
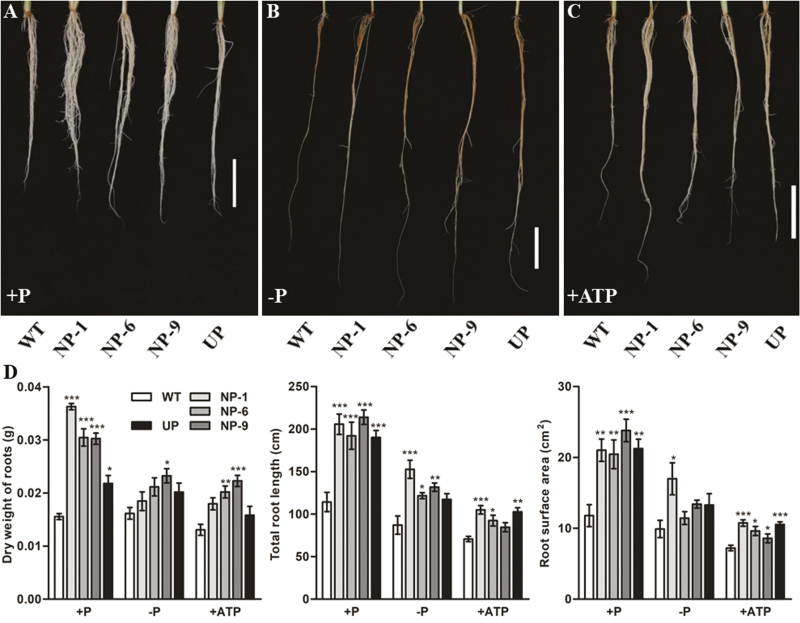
Root phenotypes and growth of wild-type (WT) rice and of transgenic plants with *OsPAP10c* driven by the constitutive ubiquitin promoter (UP) or the native promoter (NP) grown either with or without Pi, or with ATP as the only source of N. Germinated seeds were grown in normal nutrient solution for 15 d and then transferred to different solutions for 10 d. (A–C) Phenotypes of the roots under the different treatments. The scale bars are 5 cm. (D) Root dry weight, total root length, and root surface area under the different treatments. Data are means (±SEM) of six replicates. Significant differences compared with the WT were determined using Turkey’s test (*P<0.05; **P<0.01; ***P<0.001).

Growth parameters were measured in the WT, NP and UP transgenic plants under different P conditions. The shoot length and dry weight were significantly increased in the NP plants compared with the WT irrespective of the P supply (Fig. 6). The root dry weight, total root length, and root surface area were all significantly greater in the NP and UP plants compared with the WT under Pi-sufficient conditions (Fig. 7A, D), and a similar pattern was observed in the ATP treatment. Under Pi-deficient conditions, total root length was significantly increased in the NP plants but not in the UP plants. The root dry weight and surface area were only significantly affected in individual NP lines under Pi deficit (Fig. 7B, D).

**Fig. 8.**
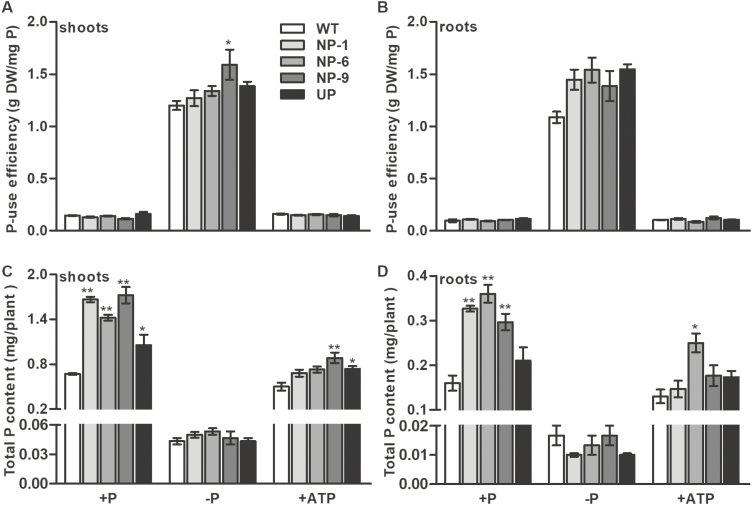
P-use efficiency of wild-type (WT) rice and of transgenic plants with *OsPAP10c* driven by the constitutive ubiquitin promoter (UP) or the native promoter (NP) grown either with or without Pi, or with ATP as the only source of P. Germinated seeds were grown in normal nutrient solution for 15 d and then transferred to different solutions for 10 d. (A, B) P-use efficiency ratio of the genotypes under the different treatments. P-use efficiency was defined as the amount of biomass produced by a given amount of P content in the tissue. (C, D) Total P content of the genotypes under the different treatments. Data are means (±SEM) of three replicates. Significant differences compared with the WT were determined using Turkey’s test (*P<0.05; **P<0.01).

We also determined Pi concentration, P-use efficiency, and P content under the different P supply conditions. Similar Pi concentrations were observed in all the genotypes in both shoots and roots irrespective of the Pi supply (Supplementary Fig. S6). The ATP treatment significantly increased the Pi concentration of the shoots in the UP plants and in one line of the NP lines. With the exception of one NP line, no significant differences in P-use efficiency were observed between the genotypes in either of the Pi treatments or the ATP treatments (Fig. 8A, B). The total P content was significantly increased in the shoots and roots of NP plants under Pi-sufficient conditions compared with the WT, but no differences were observed under Pi-deficient conditions (Fig. 8C, D). Compared with the WT, the P content of the shoots of UP plants was significantly increased under P-sufficient conditions and in the ATP treatment. The P content of the line NP-9 was also increased in the shoots and roots in the ATP treatment.

### Growth and yield of NP plants are increased under field conditions

Given the results from the hydroponic experiments, we further evaluated the performance of NP transgenic plants grown in the field with or without application of P fertilizer. Overall shoot growth and panicle size were increased in the NP plants compared with the WT (Supplementary Fig. S7). In terms of agronomical traits, similar values for tiller number, seed-setting rate, and 1000-grain weight were observed between the WT and NP plants under both low and normal P conditions (Table 1). Plant height, grain number, and grain yield per plant were significantly increased in the NP plants compared with the WT under both P treatments.

**Table 1.** Agronomic traits of wild-type and transgenic plants driven by the native promoter grown in the field under different Pi supply

Pi supply	Line	Plant height (cm)	Tiller number	Seed-setting rate (%)	1000-grain weight (g)	Grain number per plant	Grain yield per plant (g)
LP	WT	87.36±2.53^e^	26.33±3.50^a^	72.51±7.56^b^	22.56±0.36^ab^	1593.49±137.68^d^	35.95±3.26^e^
	NP-1	115.21±2.15^bc^	23.83±5.19^a^	79.91±3.04^ab^	23.91±2.09^ab^	2121.44±321.34^bc^	50.76±8.92^bcd^
	NP-6	124.64±4.62^a^	24.67±3.61^a^	79.20±5.76^ab^	23.47±1.17^ab^	1930.03±270.43^bcd^	45.48±8.16^cde^
	NP-9	113.39±3.97^c^	28.17±3.13^a^	81.50±3.12^ab^	25.01±1.95^ab^	1994.97±230.48^bcd^	49.67±5.12^bcd^
HP	WT	95.46±2.70^d^	30.17±5.49^a^	74.82±8.57^ab^	22.16±1.16^b^	1783.01±223.28^cd^	39.31±2.77^de^
	NP-1	119.36±5.00^b^	31.00±4.69^a^	83.28±2.52^a^	24.69±2.36^ab^	2818.01±433.94^a^	69.00±7.65^a^
	NP-6	124.89±4.06^a^	26.67±4.59^a^	84.37±5.77^a^	24.56±1.54^ab^	2394.53±235.01^ab^	59.00±8.42^ab^
	NP-9	113.46±4.31^c^	25.33±3.67^a^	82.58±5.58^ab^	25.49±1.61^a^	2133.21±207.66^bc^	54.22±4.24^bc^

LP, low Pi (without P fertilizer); HP, high Pi (80 kg P ha^–1^). WT, wild-type; NP, native promoter. Data are means (±SD). Different letters indicate significant differences between the WT and transgenic plants as determined by one-way ANOVA followed by Tukey’s test (*P*<0.05).

## Discussion

### *OsPAP10c* encodes a specific secreted phosphatase

It has been reported that Pi starvation induces extracellular APases, which degrade Po into Pi for plant growth and development (Tran *et al.*, 2010a). The extracellular APase proteins can be secreted into the rhizosphere or the cell wall matrix. APase activities have been measured in mutants of 29 different *PAP* genes in Arabidopsis, with the results showing that only the *Atpap12*, *Atpap15* and *Atpap26* mutants have APase activity decreased by more than 30% compared with WT plants in roots under either Pi-sufficient or -deficient conditions (Wang *et al.*, 2014). It is well established that AtPAP12 and AtPAP26 are the major APases that are secreted into the rhizosphere and cell wall in phosphate-starved Arabidopsis (Tran *et al.*, 2010b; Robinson *et al.*, 2012). However, mutations of the two corresponding genes decrease the activity of secreted APase rather than the activity APase associated with the root surface, as indicated by BCIP staining. In contrast, the *Atpap10* mutant shows a ‘white root’ phenotype and AtPAP10 has been shown to be the major root surface-associated APase in Arabidopsis (Wang *et al.*, 2011). *OsPAP10c* was previously identified as the rice homologue of *AtPAP10* and *AtPAP12*, and it is specifically induced by Pi starvation in the roots (Lu *et al.*, 2016). To determine whether OsPAP10c is one of the major secreted APase isoforms in rice, mutants were generated using CRISPR/Cas9 technology (Fig. 2A). Quantitative measurements of APase activity in the resulting mutants showed that the activity of APase secreted into the medium was decreased by more than 50% under both Pi-sufficient and -deficient conditions (Fig. 2B, C). The mutations also resulted a similar ‘white root’ phenotype in rice as seen in *Atpap10* (Fig. 2D). Moreover, *OsPAP10c* could fully complement the decreased root surface-associated APase activity in the *Atpap10* mutant (Fig. 3), indicating that OsPAP10c is an essential root surface-associated APase in rice. We then collected and analysed the proteins secreted into the medium by rice suspension cells in order to identify which APase isoforms were present (Fig. 2E). A specific isoform was absent in the samples from the *pap10c* mutant, and this might account for the significant decrease in the activity of secreted APase. Taken together, the results indicated that OsPAP10c is a major secreted APase in rice.

It has previously been shown that the major secreted APases from a variety of plant species belong to the PAP Ia family, and *OsPAP10c* is classified as being within a monocotyledon-specific PAP group (Lu *et al.*, 2016). Four homologous genes of *AtPAP10* and *AtPAP12* have been found in rice, namely *OsPAP10a*–*10d* (Zhang *et al.*, 2011; Lu *et al.*, 2016). Corresponding with this, we detected four bands in the WT plants using *OsPAP10c* as the probe in Southern blot analysis (Supplementary Fig. S3), presumably representing the homologous genes. Interestingly, these four genes show different expression patterns in rice. The expressions of *OsPAP10b* and *OsPAP10d* are extremely low and show no response to Pi starvation, whereas in contrast *OsPAP10a* and *OsPAP10c* show relatively higher expression and are induced by Pi starvation, indicating that they might be involved the adaptation of rice to low P supply (Lu *et al.*, 2016). However, expression of *OsPAP10a* is induced in both roots and leaves by Pi starvation while expression of *OsPAP10c* is only specifically induced in the roots. Although overexpression of *OsPAP10a* and *OsPAP10c* both significantly increase the utilization of Po in rice, overexpression lines of *OsPAP10c* exhibit higher activity of secreted APase compared with overexpression lines of *OsPAP10a* (Tian *et al.*, 2012; Lu *et al.*, 2016). In-gel APase activity assays have indicated that overexpression of *OsPAP10a* and *OsPAP10c* in rice increase different APase isoforms (Tian *et al.*, 2012; Lu *et al.*, 2016). We conducted a qRT-PCR analysis that ruled out the possibility that overexpression of *OsPAP10c* influenced the expression of *OsPAP10a* (Supplementary Fig. S8). Therefore, we assume that OsPAP10c represents a monocotyledon-specific APase isozyme, which can be secreted into the medium in a similar manner to AtPAP12 and can be associated with the root surface in a similar manner to AtPAP10.

### Overexpression of *OsPAP10c* enhances root growth in rice

The expression of a number of *PAP* genes are induced by Pi starvation, indicating their important roles in Pi stress responses. It is generally recognized that PAPs are involved in the degradation of extracellular Po to convert it into plant-available Pi. However, there is a growing body of evidence to show that PAPs are also involved in regulating plant growth in addition to recycling Po. Increasing numbers of PAPs are being found to be secreted into the cell wall where limited Po exists. In tobacco, NtPAP12 has been identified as a cell wall-bound APase and it regulates the wall components by dephosphorylation of α-xylosidase and β-glucosidase. Although the physiological functions of *NtPAP12* are unknown, overexpression of its homologue *AtPAP12* in Arabidopsis significantly increases plant growth under Pi-deficient conditions without extracellular Po supply (Wang *et al.*, 2014). Moreover, mutations of *AtPAP10* and *OsPAP21b* inhibit root growth in Arabidopsis and rice, respectively (Wang *et al.*, 2011; Mehra *et al.*, 2017). It has been proposed that these PAPs may regulate growth by modifying the cell wall composition. Interestingly, we found that NP transgenic plants showed higher root-associated APase activity and increased root length and surface area compared with the WT (Figs 5, 7). *OsPAP10c* may play a key function in root growth under Pi-stress conditions, which would be in accordance with the induced expression that we observed (Fig. 1). It has been reported that increasing the root length and surface area can significantly increase Pi uptake and accumulation in rice (Gamuyao *et al.*, 2012), and we found that the P content per plant was significantly increased in the NP transgenic plants compared with the WT, while the Pi concentration and P utilization efficiency did not significantly change (Fig. 8, Supplementary Fig. S6). Hence, the better performance of the NP plants may have been due to the stimulated root growth and increased nutrient uptake efficiency.

### Increased expression of *OsPAP10c* driven by its native promoter enhances plant growth and yield

Although the overexpression lines of *OsPAP10c* driven by a ubiquitin promoter showed significantly enhanced APase activity in leaves and roots, the growth of these lines is inhibited in both pot and field experiments (Lu *et al.*, 2016). Similarly, overexpression of *GmPAP1-like* and *GmPAP21* in bean hairy roots also results in a reduction in fresh weight relative to control lines under Pi-sufficient conditions (Li *et al.*, 2017; Wu *et al.*, 2018). The expression of these *PAP* genes was induced by Pi starvation in specific tissues, whereas their constitutive overexpression would increase APase activity and degrade organic P compounds in all tissues included untargeted ones, and this might cause negative effects in the transgenic plants. To try to avoid this, we overexpressed *OsPAP10c* in rice by using its native promoter. As expected, the NP transgenic plants had increased expression of *OsPAP10c* to a moderate level, which was significantly lower than that in the UP transgenic plants (Fig. 4), and accordingly the APase activity was not changed in the leaves and was slightly increased in roots (Supplementary Fig. S5). Interestingly, the activities of both root surface-associated and secreted APase were significantly increased in the NP plants compared with the WT, although they were still lower than in the UP plants.

To evaluate the effects of overexpressing *OsPAP10c* by use of its native promoter, the transgenic plants were grown under different Pi supply conditions. Compared with the WT and UP plants, the growth of NP plants was significantly increased irrespective of the Pi supply (Fig. 6). This indicated that overexpression of *OsPAP10c* by its native promoter rather than a constitutive promoter was an effective way to stimulate plant growth. However, the expression levels and APase activity were not tightly correlated with the copy number of *OsPAP10c*. The three lines with the best performance (NP-1, NP-6, and NP-9) showed a level of expression level that was comparable with some of the other lines (Fig. 4). This variation in expression and phenotype between the different transgenic lines may have been be influenced by different insertion positions of the T-DNA or by random mutations during the tissue culture process in addition to copy number. Another possibility is that post-transcriptional regulation of *OsPAP10c* exists. Although the expression of *PAP* genes can be increased by a factor of tens or hundreds, in all the studies to date the activity of APase was always been increased only by a few fold (Wang et al., 2011, 2014; Tian *et al.*, 2012; Li *et al.*, 2017; Wu *et al.*, 2018). Thus, the difference in APase activity is several magnitudes smaller than the difference in transcript levels in the plants, which would accounts for the similar APase activities in the different transgenic lines.

We conducted a field experiment to examine the agronomic traits of the NP transgenic plants, and found that plant growth and grain yield were significantly enhanced under both low and normal P conditions (Table 1). Overall, our results indicate that using the native promoter to overexpress PAPs that are induced by Pi starvation provides a potential means to improve future crop production

## Supplementary data

Supplementary data are available at *JXB* online.

Fig. S1. Time-course of expression levels of *OsPAP10c* in leaves and roots under different P treatments, as extracted from previously published RNA-seq data.

Fig. S2. Acid phosphatase activities in leaves and roots of the wild-type and *pap10c* mutants under different Pi supply conditions.

Fig. S3. Southern blot analysis of the wild-type and transgenic plants using the *hpt* and *OsPAP10c* probes.

Fig. S4. Phenotypes and fresh weights of the wild-type and transgenic plants under different Pi supply conditions.

Fig. S5. Acid phosphatase activities in the leaves and roots of the wild-type and transgenic plants under different Pi supply conditions.

Fig. S6. Pi concentrations in the shoots and roots of the wild-type and transgenic plants under different Pi supply conditions, and with supply of ATP.

Fig. S7. Phenotypes of shoots and panicles of the wild-type and NP transgenic plants grown in the field with or without P fertilizer.

Fig. S8. The expression of *OsPAP10a* in the wild-type and *OsPAP10c* NP transgenic plants under different Pi supply conditions.

eraa179_suppl_Supplementary_Figures

## Abbreviations

APase: acid phosphatase
Pi: phosphate
Po: organic phosphate
NP: native promoter
UP: ubiquitin promoter
