# Supplementary material for: Purple acid phosphatase 10c encodes a major acid phosphatase that regulates plant growth under phosphate-deficient conditions in rice
Source: J Exp Bot. 2020 Apr 9;71(14):4321–32. doi: 10.1093/jxb/eraa179 (PMC7475256; doi:10.1093/jxb/eraa179)
Supplement: eraa179_suppl_Supplementary_Figures [file eraa179_suppl_supplementary_figures.pdf]

Figure S1

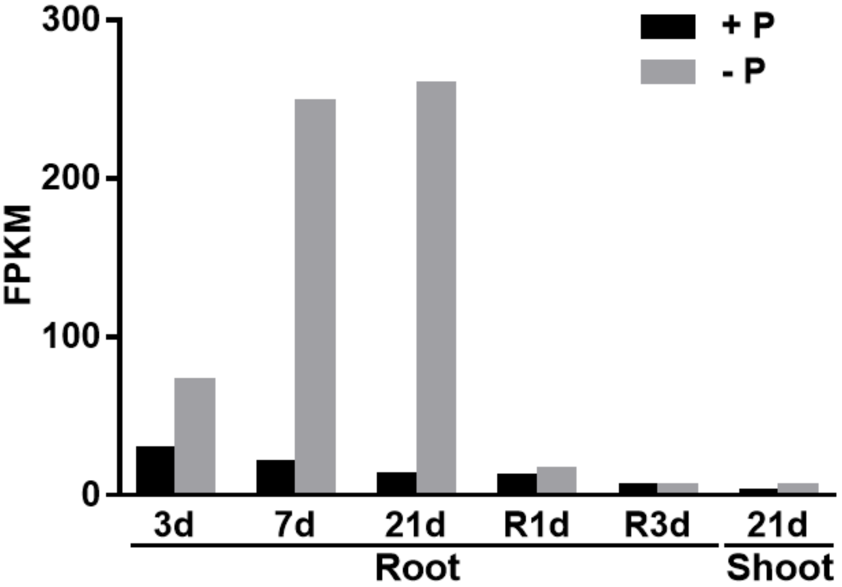

Figure S1. The expression of *OsPAP10c* in roots and shoot under Pi sufficient and deficient conditions.

Figure S2

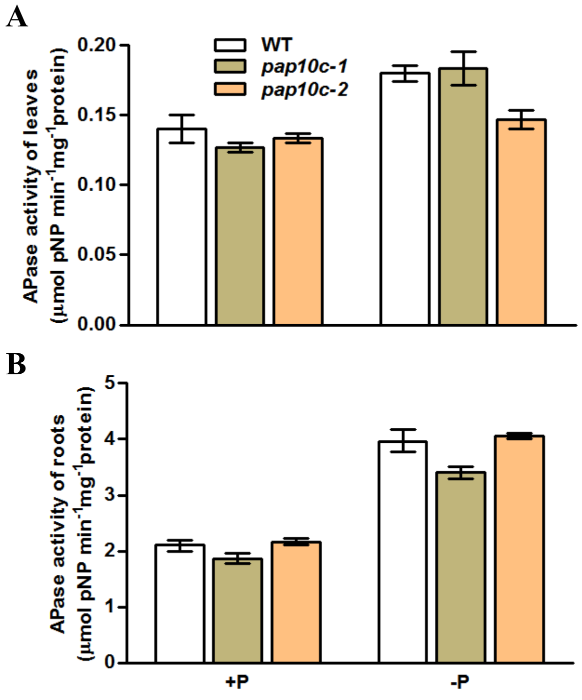

Figure S2. Acid phosphatase activities in leaves and roots of wild type (WT) and *pap10c* mutants.

Figure S3

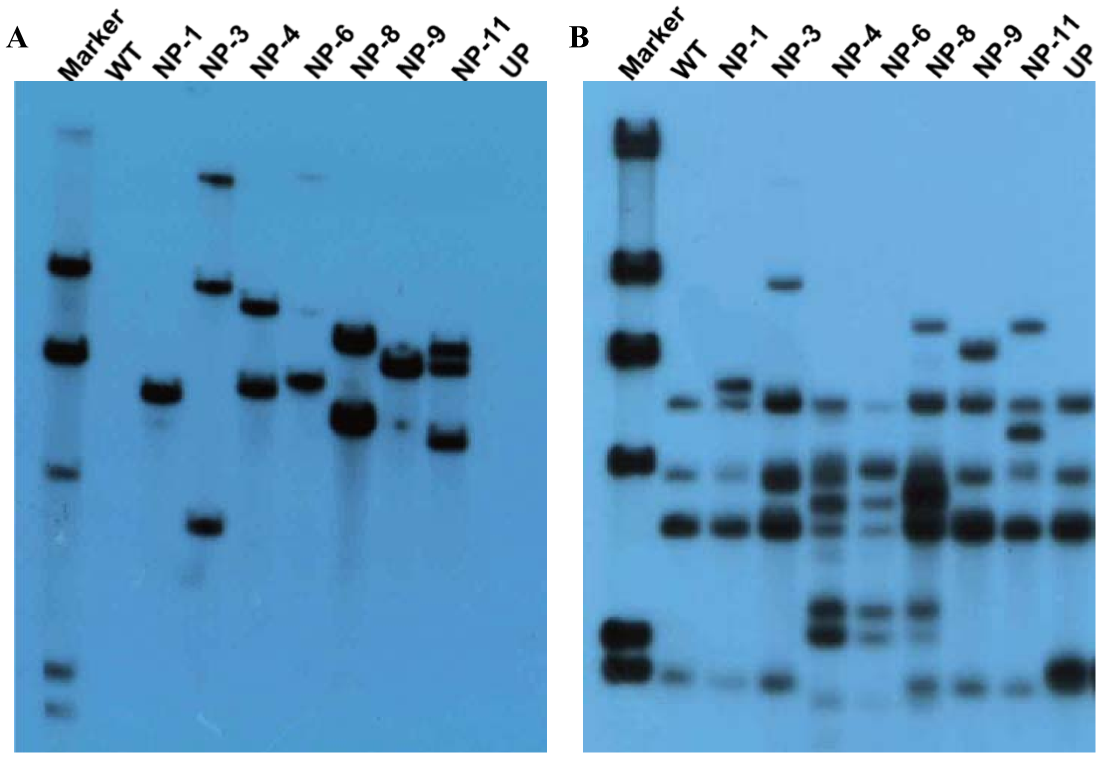

Figure S3. Southern blot analysis of WT and transgenic plants.

Figure 4

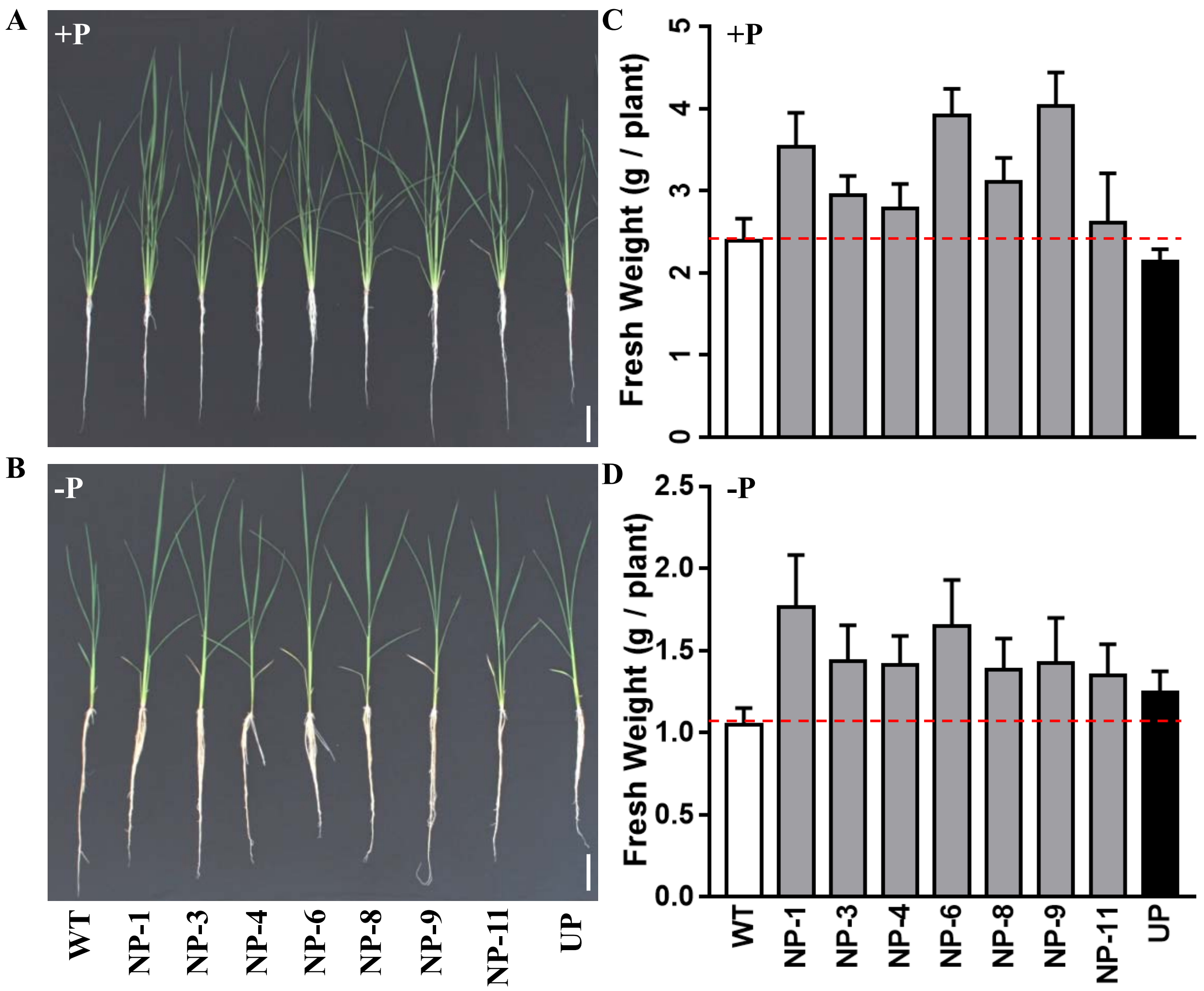

Figure S4. Phenotype of WT and transgenic plants under Pi sufficient and deficient conditions.

Figure S5

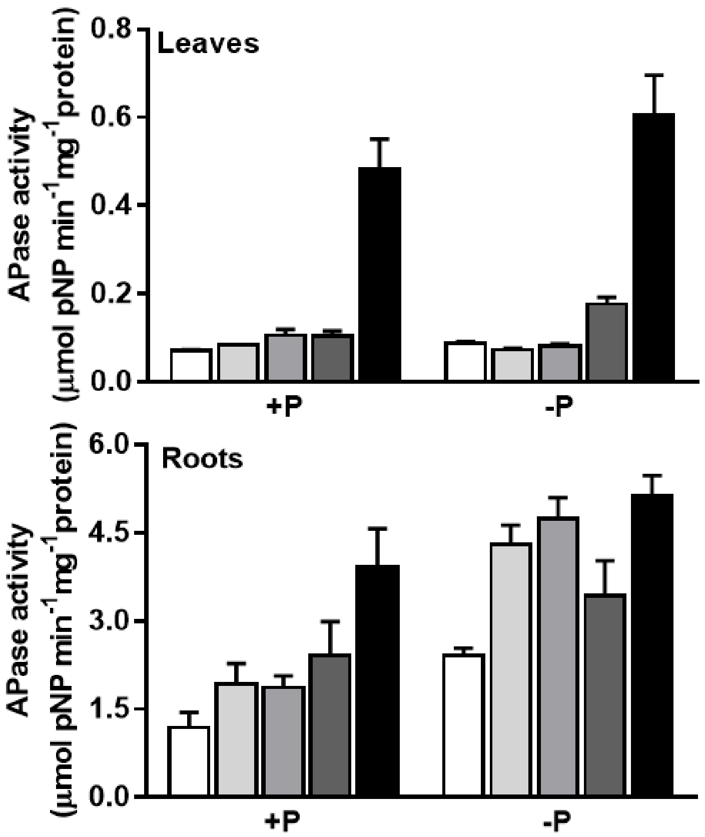

Figure S5. Acid phosphatase activities in leaves and roots of WT and transgenic plants under Pi sufficient and deficient conditions.

Figure S6

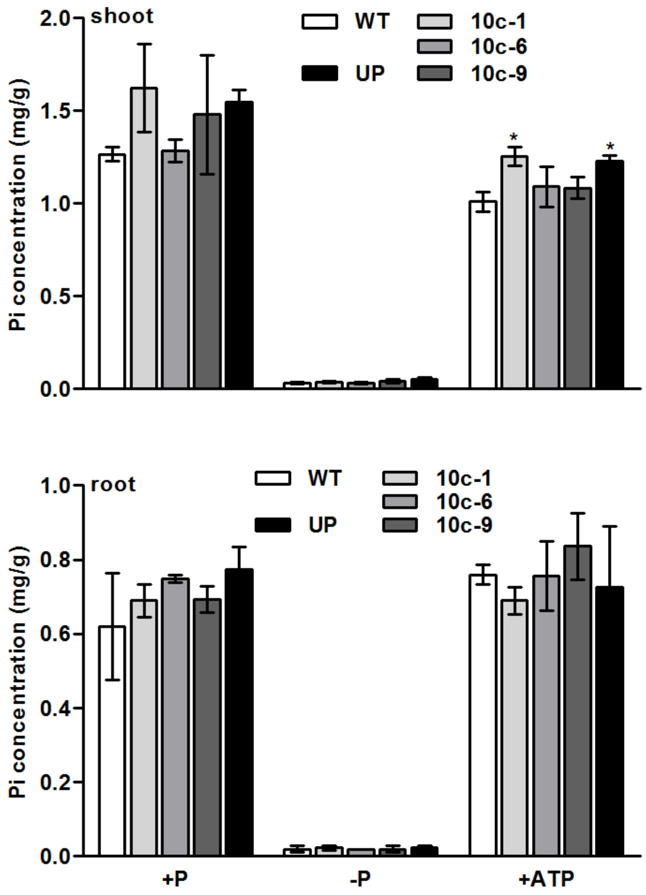

Figure S6. Pi concentration of WT and transgenic plants under Pi sufficient, deficient and ATP supply conditions.

Figure S7

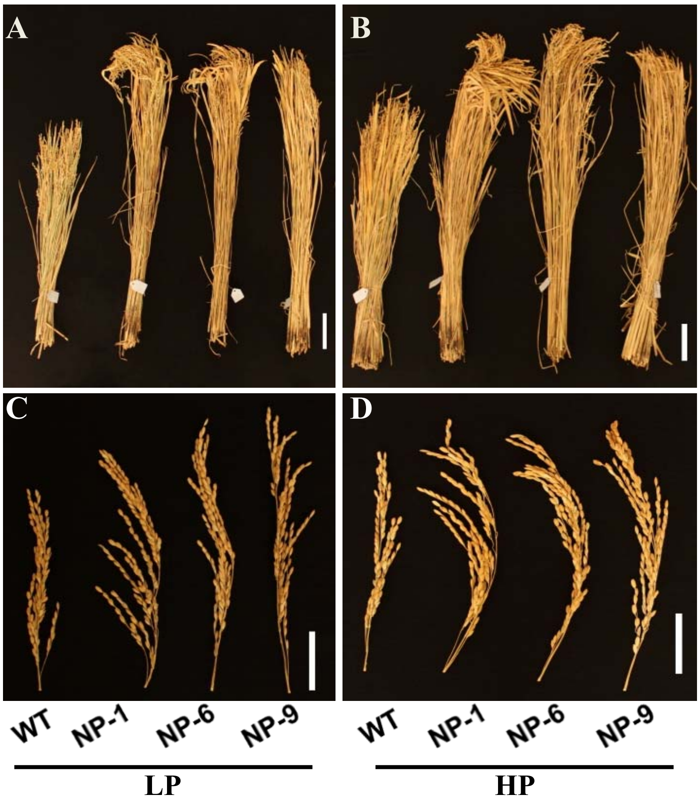

Figure S7. Growth performance and panicle size of WT and transgenic plants grown in the field, Bar = 10 cm.

Figure S8

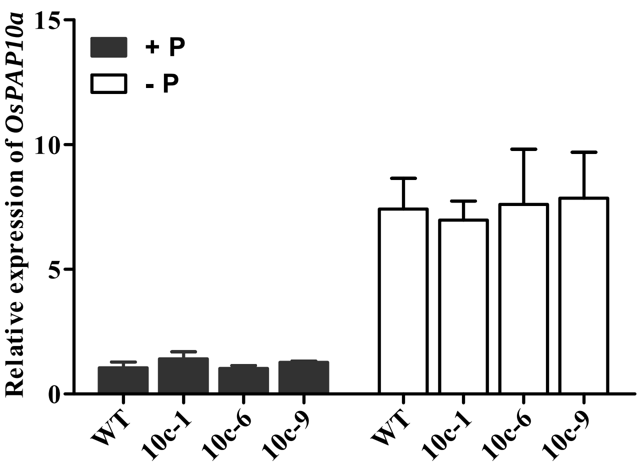

Figure S8. The expression of *OsPAP10a* in WT and *OsPAP10c* NP transgenic plants under Pi sufficient (+P) and deficient (-P) conditions.
